# Food selectivity and eating difficulties in adults with autism and/or ADHD

**DOI:** 10.1177/13623613251314223

**Published:** 2025-02-25

**Authors:** Sarah C. Bayoumi, Ashley Halkett, Meghan Miller, Stephen P. Hinshaw

**Affiliations:** 1 University of California, Berkeley, USA; 2 University of California, Davis, USA; 3 University of California, San Francisco, USA

**Keywords:** ADHD, adults, autism, eating, feeding, insistence on sameness, sensory sensitivity

## Abstract

Children with autism or attention-deficit/hyperactivity disorder (ADHD) are more likely than neurotypical children to experience eating difficulties, such as picky/selective eating, slow eating, and over/under-eating. Recent studies show that eating difficulties can persist into adulthood for autistic individuals. Yet little is known about the mechanisms involved and whether adults with ADHD also experience such challenges. We (1) compare eating difficulties among adults with autism, ADHD, autism/ADHD, and neither condition and (2) identify predictors of overall eating pathology and of food selectivity. The sample was 50% female and 78% White, consisting of 961 adults aged 19-57 who self-reported their diagnostic status: 184 with autism, 416 with ADHD, 292 with autism/ADHD, and 69 comparisons. Eating difficulties were assessed via self-report survey using the Swedish Eating Assessment for Autism (SWEAA) and Food Preferences Questionnaire. Autistic adults, with or without ADHD, reported significantly more eating difficulties than all other groups. Total eating difficulties were associated with increased insistence on sameness, other autistic features, food selectivity, and hyperactivity-impulsivity. Food selectivity was predicted by increased insistence on sameness, sensory sensitivity, and other core features of autism, and lower hyperactivity-impulsivity. The high degree of eating difficulties in autistic adults warrants clinical attention on this topic.

**Lay Abstract:**

Some people do not like many foods, eat very quickly or slowly, or eat too much or too little. These problems are more common in children with autism or ADHD and may continue for a long time, but we do not know much about these problems in adults. Our goal was to understand how eating difficulties are similar and different in adults with autism, ADHD, both autism/ADHD, and neither condition. We also wanted to understand factors that relate to picky eating and overall eating difficulties. We found that autistic adults had the most problems with eating compared to all other groups. Adults with ADHD had more eating problems than adults with neither condition. Adults with autism and/or ADHD were more sensitive to taste and texture of foods, had difficulty with spilling food, and found it hard to tell whether they are hungry or full compared to people without these conditions. We also found that autism and ADHD traits were linked to picky eating and having more eating problems. The results show that many autistic adults and some with ADHD might need support with eating. Doctors should pay attention to eating problems to help people get the care they need. Results also show that wanting things to stay the same may be more related to eating problems than researchers previously thought. We need more research to understand how to support adults with eating difficulties.

Autism spectrum disorder (autism^1^) is a lifelong neurodevelopmental condition characterized by social-communication challenges and restricted, repetitive behaviors (American Psychiatric Association [APA], 2022). Many autistic individuals experience challenges with eating, such as food selectivity (picky/selective eating), oral sensory sensitivity, rigid mealtime routines, and restrictive eating (Baraskewich et al., 2021). Indeed, autistic children are five times more likely to exhibit eating difficulties than non-autistic peers (Sharp, Berry, et al., 2013), and as many as 88% eat a limited range of food (Cermak et al., 2010; Dominick et al., 2007). Autistic children tend to prefer refined carbohydrates (e.g. white bread) and bland foods (e.g. chicken nuggets; Mayes & Zickgraf, 2019); some refuse to eat vegetables (Sharp, Jaquess and Lukens, 2013). These concerns are associated with increased psychosocial stress and health problems like gastrointestinal issues, malnourishment (e.g. calcium and protein deficits), unhealthy weight, internalizing problems, and avoidance of social situations involving food (Bandini et al., 2017; Crippa et al., 2022; Leader et al., 2020; Sharp, Berry, et al., 2013; Thullen & Bonsall, 2011). Caregiver reports indicate that some such behaviors may begin as early as 6 months of age (Dominick et al., 2007), with more striking differences apparent by 15 months (Emond et al., 2010; Mayes & Zickgraf, 2019). Of particular concern, eating difficulties can extend into adulthood, which is a developmental period distinguished by greater independence and additional demands (Bandini et al., 2017; Nisticò et al., 2022). While children look to their caregiver(s) to provide food, adults living independently or with reduced support need to balance food purchase, preparation, and healthy decision-making about food intake. Furthermore, eating plays an important role in adult social practices, as many community events, workplace functions, and dating experiences involve food to some degree. For those with social and executive functioning challenges, these added mealtime demands might exacerbate eating difficulties and the negative impacts in adulthood.

Recent evidence suggests that certain eating difficulties may also be associated with attention-deficit/hyperactivity disorder (ADHD), a neurodevelopmental condition involving challenges with inattention and/or hyperactivity-impulsivity across multiple settings (APA, 2022). Autism and ADHD share several common presumed etiological factors (e.g. genetic variants) and developmental pathways (e.g. elevated rates of ADHD in later-born siblings of children with autism; Miller et al., 2019; Rommelse et al., 2010; Ronald et al., 2017; Stergiakouli et al., 2017). These conditions also frequently co-occur and share behavioral overlap, such as social-communication and sensory differences (Dellapiazza et al., 2021; Leitner, 2014; Stevens et al., 2016). Like autistic individuals, children with ADHD experience significantly more food selectivity, sensory sensitivity, and disruptive mealtime behaviors than neurotypical (NT) children (Mayes & Zickgraf, 2019; Sha’ari et al., 2017). Approximately 17% of children with ADHD display atypical eating behaviors, in contrast to 4.8% of NTs (Mayes & Zickgraf, 2019). Other documented eating difficulties in individuals with ADHD include greater consumption of energy-dense, nutrient-poor foods; binge eating; overeating; and purging (Kaisari et al., 2017; Wang et al., 2019). Furthermore, hyperactivity-impulsivity symptoms of ADHD have been associated with increased eating difficulties in children (Råstam et al., 2013; Zucker et al., 2015). To our knowledge, food selectivity—the most common eating concern in both children with ADHD and children with autism (Margari et al., 2020; Mayes & Zickgraf, 2019)—has not been studied in adult ADHD samples, making it unclear whether this population is also at risk of eating difficulties as adults.

Investigations of autistic individuals suggest that eating difficulties observed in childhood may not fully resolve by adulthood. Over 6 years, Bandini et al. (2017) found a significant decrease in “problematic mealtime behaviors” (e.g. fidgeting while eating) but an increase in overweight/obese status, from a mean age of 6.9 to 13.2 years. Even more, half of autistic children remained high in food selectivity, and two-thirds had even more limited food repertoires at follow-up. In addition, two cross-sectional studies of autistic adolescents documented a positive association between age and obsessive dietary rituals; consumption of energy-dense, nutrient poor foods; and unhealthy weight (Mathew et al., 2022; Park et al., 2021), suggesting that certain eating difficulties may persist (or arise) beyond childhood. In the limited literature on autistic adults, findings show similar challenges with food selectivity and mealtime rituals (Kuschner et al., 2015; Spek et al., 2020). Yet most of the adult eating literature, especially on ADHD, focuses on “traditional” disordered eating motivated by a preoccupation with weight or body shape (Kaisari et al., 2017), making it unclear how to effectively tailor food selectivity interventions to adults, who possess greater control over their own food intake than children.

Only a handful of studies have directly compared eating behaviors in individuals with autism or ADHD. It appears that children with autism or ADHD are significantly more likely than NTs to exhibit eating difficulties, with autistic children experiencing these concerns at the highest rate (Mayes & Zickgraf, 2019; Råstam et al., 2013). Autism and ADHD diagnoses are also related to increased eating challenges in adults (Remnélius et al., 2022). Little research has examined eating in co-occurring autism/ADHD, likely complicated by the historical prohibition against dual diagnosis of these conditions. The limited research suggests that children with elevated autism/ADHD features (or a concurrent diagnosis) show greater total eating difficulties than children with autism-only, ADHD-only, or neither condition (Harris et al., 2022; Råstam et al., 2013; for conflicting findings, see Nadon et al., 2011). There is a need to transcend measures of overall eating difficulties by focusing on specific kinds of eating problems.

One possible mechanism underlying eating difficulties in autism and ADHD is sensory sensitivity—a finding supported in autism-specific samples, ADHD-specific samples, and general population studies (Crippa et al., 2022; B. Smith et al., 2020; Kirby et al., 2022; Zucker et al., 2015). Sensory sensitivity was added to the diagnostic criteria for autism in the *DSM*-5 and is also estimated to affect 50% to 70% of children with ADHD (Dellapiazza et al., 2021; Mimouni-Bloch et al., 2018; Rani et al., 2023). Sensory sensitivity presents in several forms, including hyper- or hypo-reactivity to input from the environment or unusual sensory-seeking interest across modalities like taste and smell (APA, 2022). In particular, texture and consistency of food are reported by parents as primary reasons children with and without autism reject food (Hubbard et al., 2014).

Insistence on sameness, another core feature of autism under the *DSM-5* restricted and repetitive behavior (RRB) domain, may also be a focal reason for at least some of the described eating difficulties noted earlier (Suarez & Crinion, 2015; Zickgraf et al., 2022). It is characterized by the need for consistent routines and rituals, resulting in severe distress after any deviation (APA, 2022). Insistence on sameness could be a coping mechanism that allows autistic individuals to reduce uncertainty, gain a sense of control in their environment, and/or avoid unpleasant sensory stimuli (Park-Cardoso & da Silva, 2022). Many autistic individuals have strict mealtime routines, such as not allowing different foods to be touched or sitting in the same seat at every meal (Mayes & Zickgraf, 2019; Suarez & Crinion, 2015). Autistic children may also have strong preferences for the brand, shape, and presentation of food, refusing to consume alternative options (Hubbard et al., 2014). These behaviors are often considered examples of insistence on sameness. Still, it can be difficult to disentangle the extent to which such behaviors are related to a need for sameness versus sensory preferences of taste and texture.

In all, the gap in the literature is threefold: (a) Research on food selectivity and other eating difficulties rarely includes adults, (b) little is known about shared versus distinct eating difficulties in autism and ADHD, and (c) much remains unclear about how core features of these conditions affect eating behavior. Given that daily living skills, support needs, and features of these conditions can shift over the lifespan (Hartman et al., 2016), it is important to examine how and whether eating difficulties common in childhood manifest in adulthood. Identifying specific eating challenges and their mechanisms could help inform clinical services and supports. Overall, we seek to (1) compare eating difficulties among adults with autism, ADHD, co-occurring autism/ADHD, and neither condition; and (2) identify predictors of both food selectivity and total eating difficulties. We hypothesize that adults with autism and/or ADHD will have more eating difficulties than comparison participants and that autistic adults will have greater eating difficulties than those with ADHD. We also hypothesize that eating difficulties and food selectivity will be predicted by increased insistence on sameness, sensory sensitivity, and hyperactivity-impulsivity.

## Method

### Participants

A total of 1,517 individuals completed the survey, which included an online screening questionnaire for prior diagnoses of autism, ADHD, eating disorders, or any other psychological disorders. Participants without formal diagnoses of autism and/or ADHD were asked if they self-identify or suspect having either of these conditions. The clinical diagnostic groups included only those with a self-reported professional diagnosis. The comparison group consisted of participants who (1) reported no professional diagnoses of autism and ADHD and (2) do not self-diagnose with autism or ADHD. Exclusion criteria entailed being outside the age range of 18-59 years old or unable to read English. We excluded 485 participants from analyses due to not meeting inclusion/exclusion criteria; having completed the survey in under 10 min; or being flagged as a Bot by Qualtrics. This yielded a final sample of 961 participants. Demographic data are presented in Table 1.

**Table 1.** Demographic data of sample.

		Autism (*n* = 184)	ADHD (*n* = 416)	Autism/ADHD (*n* = 292)	Comparison group (*n* = 69)	Total sample (*N* = 961)	*p*
Gender (n,%)							***
	Men	33 (17.93)	42 (10.10)	37 (12.67)	18 (26.09)	130 (13.53)	
	Women	101 (54.89)	316 (75.96)	162 (55.48)	47 (68.12)	626 (49.84)	
	Non-binary	50 (27.17)	58 (13.94)	93 (31.85)	4 (5.80)	205 (21.33)	
Mean age in years (*SD*)		28.82 (8.16)	29.70 (7.03)	28.84 (7.87)	32.06 (11.89)	29.44 (7.97)	*
Race (n,%)							***
	Asian	3 (1.63)	16 (3.85)	5 (1.71)	15 (21.74)	39 (4.06)	
	Black or African American	1 (0.54)	3 (0.72)	7 (2.40)	1 (1.45)	12 (1.25)	
	Mixed Race or Multiracial	34 (18.48)	54 (12.98)	50 (17.12)	10 (14.49)	148 (15.4)	
	Other	3 (1.63)	4 (0.96)	6 (2.05)	2 (2.9)	15 (1.56)	
	White	143 (77.72)	339 (81.49)	224 (76.71)	41 (59.42)	747 (77.73)	
Ethnicity (n, %)							***
	Hispanic or Latino	13 (7.07)	14 (3.37)	7 (2.40)	23 (6.32)	57 (4.54)	
	Not Hispanic or Latino	171 (92.93)	402 (96.63)	285 (97.60)	341 (93.68)	1199 (95.46)	
Educational Attainment (n, %)							***
	Some high school or less	74 (40.22)	211 (50.72)	101 (34.59)	38 (55.07)	424 (44.12)	
	High school diploma or GED	29 (15.76)	66 (15.87)	52 (17.81)	3 (4.35)	150 (15.61)	
	Some college, but no degree	20 (10.90)	32 (7.69)	35 (11.99)	6 (8.70)	93 (9.68)	
	Associate’s or technical degree	6 (3.26)	22 (5.29)	18 (6.16)	3 (4.35)	49 (5.10)	
	Bachelor’s degree	37 (20.11)	70 (16.83)	46 (15.75)	17 (24.64)	170 (17.69)	
	Graduate or professional degree	1 (0.54)	2 (0.48)	0 (0.00)	2 (2.90)	5 (0.52)	
	Prefer not to say	17 (9.24)	13 (3.13)	40 (13.70)	0 (0.00)	70 (7.28)	
Mental Health Diagnosis (n, %)	Eating disorder	39 (21.20)	68 (16.35)	73 (25.00)	5 (7.25)	185 (19.25)	**
	Other mental illness	153 (83.15)	316 (76.14)	246 (84.25)	24 (34.78)	739 (76.98)	***
Currently taking medication affecting appetite (n, %)		20 (5.12)	221 (56.52)	117 (29.92)	4 (5.80)	362 (37.67)	***
Currently on a diet (n, %)		19 (10.33)	36 (8.65)	25 (8.59)	9 (13.04)	89 (9.27)	0.62

* *p* < .05. **p < .01. ***p < .001.

### Procedures

All study procedures were approved by the Institutional Review Board at UC Berkeley. Participants were recruited through digital flyers and postings in social media channels that people with autism and/or ADHD may be more likely to frequent, including Reddit, Instagram, and TikTok. A snowball sampling approach was employed, wherein participants were encouraged to send information about the study to other potential participants. Recruitment materials contained information that the study was being led by a team of autistic and non-autistic researchers. We aimed to make the sample as representative of the autism and ADHD populations as possible by prioritizing a large sample size given the heterogeneous nature of these conditions.

All recruitment materials directed participants to a survey link. Written informed consent was required to gain access to the study questionnaires via Qualtrics. After completing the surveys, participants were provided with a list of resources if they wished to seek support for their eating. Participants were not compensated.

### Measures

#### Swedish Eating Assessment for Autism Spectrum Disorders

The Swedish Eating Assessment for Autism Spectrum Disorders (SWEAA) is a self-report questionnaire developed for autistic adolescents and young adults that examines eating behaviors (Karlsson et al., 2013). To our knowledge, it is the only eating questionnaire validated in autistic and non-autistic adults up to age 25 (Karlsson et al., 2013). We used the full SWEAA containing 65 items, which includes 8 subscales. The subscales include the following: Perception (oral sensory sensitivity, α = 0.83), Motor Control (messy eating, α = 0.77), Purchase of Food (brand preferences, α = 0.64), Eating Behavior (rigid food repertoire, α = 0.68), Mealtime Surroundings (routines and rituals, α = 0.90), Social Situation at Mealtime (eating in public, α = 0.64), Other Behavior Associated with Disturbed Eating (disordered eating, α = 0.68), and Hunger/Satiety (hunger cues, α = 0.55). Each item is answered on a five-point Likert-type scale ranging from 0 (*never correct*) to 4 (*strongly correct*). Because the SWEAA was developed in Sweden, we reworded some items slightly to better suit American culture and grammar.

Total and subscale scores were calculated for the SWEAA. Scoring procedures were consistent with the descriptions of Karlsson et al. (2013), involving taking the mean across the first 60 items, and then multiplying that value by 25, yielding a range of 0 to 100. Higher scores denote greater total eating difficulties. Subscale scores were similarly computed. Missing responses were imputed by calculating the mean with an adjusted denominator corresponding to the number of completed items.

#### Food Preferences Questionnaire for Adolescents and Adults

The Food Preferences Questionnaire (FPQ) is a self-report measure that assesses food selectivity (A. D. Smith et al., 2016). Individuals were asked to rate how much they liked a certain food on a five-point scale ranging from *not at all* to *a lot*. If participants had not tried a specific food before, they were instructed to respond “not applicable/unknown.” Psychometric properties provided by the original author suggested good validity and reliability (Smith, personal communication, March 28, 2023).

We adapted the FPQ because of findings that certain foods would be particularly relevant to our sample. First, we incorporated items from the FPQ for Children (Fildes et al., 2014), which contains items of particular interest that are not included in the FPQ for Adolescents and Adults, such as chicken nuggets and pasta. Then, we added additional food group categories including desserts (e.g. cake), spices (e.g. cinnamon), and others (e.g. pizza). These changes were based on findings that autistic children prefer bland foods, sweets, and specific meals such as chicken nuggets and pizza (Mayes & Zickgraf, 2019; Schreck & Williams, 2006). Finally, we modified UK-specific food names to make them more relevant to the American population (e.g. “crisps” changed to “chips”). The adapted scale included 61 food items and had good internal consistency (α = 0.88). A total food selectivity score was calculated by taking the meaning of all answered items. “Not applicable/unknown” responses were re-coded as missing, per the original author’s instructions (A. D. Smith et al., 2016). Missing responses were handled as described by the SWEAA.

#### Adult Repetitive Behaviors Questionnaire-2

The Repetitive Behaviors Questionnaire-2 (RBQ-2A) is a self-report measure of restricted and repetitive behaviors in adults resembling the *DSM-5* autism criteria. The RBQ-2A has two factors: Repetitive Motor Behaviors and Insistence on Sameness (Barrett et al., 2015). The eight-item Insistence on Sameness subscale was administered to capture this trait outside of the context of eating (α = 0.80); it was scored by calculating the mean of all three-point items (Barrett et al., 2015). Missing responses were accounted for by adjusting the denominator when taking the mean.

#### Autism Spectrum Quotient

The Autism Spectrum Quotient (AQ) is a 50-question self-report questionnaire validated to measure traits of autism in adults (Baron-Cohen et al., 2001). Participants responded on a four-point Likert-type scale ranging from *definitely disagree* to *definitely agree*. To minimize construct overlap with the insistence on sameness measure from the RBQ-2A, items 2, 25, 30, 34, and 43 were removed from the AQ total score. Total scores of other autistic features were computed using the original author’s guidelines (Baron-Cohen et al., 2001). The scale had good internal consistency in the current study (α = 0.88).

#### Swanson, Nolan, and Pelham Rating Scale-IV

The Swanson, Nolan, and Pelham Rating Scale-IV (SNAP-IV) assessed for core features of ADHD, namely, inattention and hyperactivity-impulsivity (Swanson et al., 2001). It has been validated and deemed reliable in a clinical sample of individuals with ADHD (Bussing et al., 2008). We used the abbreviated version of the SNAP-IV from the MTA Study containing 18 items of ADHD symptoms and adapted it as a self-report measure (Swanson et al., 2001). The inattention and hyperactivity-impulsivity subscales had good internal consistency (α = 0.89 and 0.86, respectively) and were used to measure ADHD symptoms in this study.

### Community involvement

The study was conducted by a neurodiverse team of researchers. In addition, we integrated the various perspectives of adults with autism and/or ADHD into our research design through five focus groups on this topic. The student-run autism organization at UC Berkeley, composed primarily of autistic adults and family members of people with autism, assisted with recruitment by promoting the survey link.

### Data analytic plan

Data were analyzed in *R* (script available upon request). Sociodemographic differences across groups were assessed using chi-Square tests and analysis of variance. Preliminary analyses showed a significant skew in eating difficulty scores, so data were winsorized. All subsequent analyses were performed using the winsorized data.

First, analysis of covariance was used to evaluate the difference in total SWEAA scores across diagnostic groups. Tukey’s HSD test was conducted for post hoc comparisons between groups. Multivariate analysis of variance was used to examine group differences in subscale scores on the SWEAA, with Bonferroni correction for post hoc analyses.

Next, we conducted hierarchical multiple regression analyses to examine predictors of total SWEAA scores. The first model included covariates known to influence eating behavior: gender (women vs non-women and men vs non-men), age, history of an eating disorder diagnosis (present at any point during lifetime vs absent), presence of any other mental illnesses, and current use of medication affecting appetite (e.g. stimulants for ADHD). The medication covariate included medications with either appetite promotion or suppression effects; however, 88% of participants taking medication reported that their medication suppresses their appetite. The second model added potential predictor variables of interest measured by the RBQ-2A, AQ, SNAP-IV, and FPQ: insistence on sameness, other autistic features, inattention, hyperactivity-impulsivity, and food selectivity.

Finally, we conducted hierarchical multiple regression to identify variables associated with food selectivity on the FPQ. The first model contained the same covariates as the preceding analysis. The second model added variables of interest via subscales on the SWEAA, AQ, and SNAP-IV: perception, purchase of food, eating behavior, mealtime surroundings, other autistic features, inattention, and hyperactivity-impulsivity. We compared regression models using the adjusted likelihood ratio test. As a sensitivity analysis, regressions were reconducted after scoring the AQ with all 50 items, allowing us to determine the role of insistence on sameness on eating difficulties over and above other features of autism.

## Results

### Diagnostic group differences in eating difficulties

**Figure 1.**
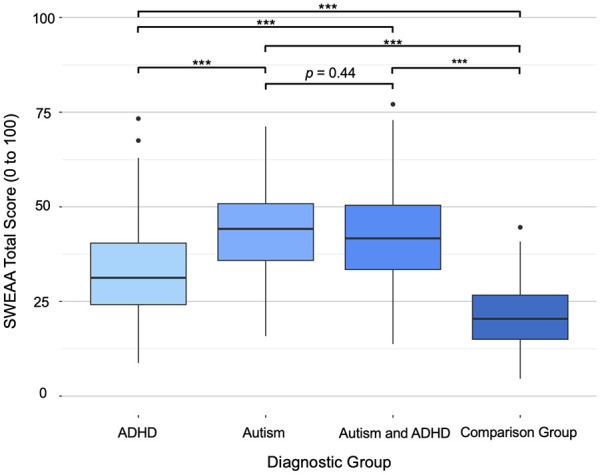
Total SWEAA scores by diagnostic group. ***p < .001.

One-way ANCOVA examined group differences in total eating difficulties. Total SWEAA scores differed significantly by a diagnostic group after adjusting for covariates, *F*(3,936) = 78.10, *p* < 0.001. Adults with autism-only had the highest mean score for eating problems (*M* = 43.8, *SD* = 11.0), followed by those with autism/ADHD (*M* = 42.3, *SD* = 11.7), adults with ADHD-only (*M* = 32.4 *SD* = 11.5), and comparisons (*M* = 21.3, *SD* = 8.99). Post hoc analyses indicated significant differences in total SWEAA scores between all groups (all *p*s < 0.001) with the exception of the autism vs autism/ADHD contrast (*p* = 0.44; see Figure 1 and Table 2). Demographics data are available by diagnostic group in Table 1.

**Table 2.** Tukey’s HSD post hoc analysis of total SWEAA scores by diagnostic group.

		Mean difference	95% CI		*p*	Cohen’s d
			LL	UL		
Autism	Autism–ADHD	11.42	8.92	13.93	***	1.19
	Autism–Comparison	22.49	18.58	26.41	***	0.53
	Autism–Autism/ADHD	1.49	1.17	4.15	0.48	0.74
ADHD	ADHD–Comparison	11.07	7.45	14.69	***	0.97
	ADHD–Autism/ADHD	-9.94	-12.10	-7.78	***	1.54
Autism/ADHD	Autism/ADHD–Comparison	21.001	17.28	24.73	***	0.72

CI = confidence interval; LL = lower limit; UL = upper limit.

***p < .001.

For the covariates, we found significant effects for gender (*F*(1, 936) = 28.87, *p* < 0.001), such that non-binary individuals scored highest on total eating difficulties, followed by females, then males (all *ps* < 0.01). There was a significant effect for eating disorder diagnosis (*F*(1, 936) = 46.89, *p* < 0.001) such that individuals with a lifetime history of an eating disorder exhibited more total eating difficulties than those without. We also found a significant effect for age (*F*(1, 936) = 33.58, *p* < 0.001). There were no significant effects for other mental illness (*F*(1, 936) = 0.70, *p* = 0.40), medication use (*F*(1, 936) = 0.12, *p* = 0.73), race (*F*(4, 936) = 1.30, *p* = 0.27), nor ethnicity (*F*(1, 936) = 0.08, *p* = 0.78).

### Specific types of eating problems by diagnosis

One-way MANOVA was used to analyze group differences in SWEAA subscale scores. A significant multivariate effect for diagnostic groups was found, Pillai’s Trace = 0.20, *F*(24, 2826) = 15.41, *p* < 0.001. Thus, Bonferroni-corrected post hoc analyses were performed to examine eating problems at the subscale level. See Table 3 for all subscale scores by group.

**Table 3.** SWEAA subscale scores by diagnostic group.

Subscales	Comparison group (*n* = 69) (A)	ADHD (*n* = 416) (B)	Autism (*n* = 184) (C)	Autism/ADHD (*n* = 292) (D)	*p*	Effect size (Partial eta squared)	Comparison
Perception, Mean, (*SD*)	24.9 (16.6)	41.7 (17.1)	56.4 (15.0)	54.4 (16.9)	***	0.22	A < B A < C A < D B < C B < D
Motor Control	14.8 (10.8)	27.0 (16.0)	30.2 (17.4)	32.3 (17.2)	***	0.07	A < B A < C A < D B < D
Purchase of Food	46.9 (19.3)	54.0 (21.1)	67.6 (21.1)	65.3 (22.0)	***	0.09	A < C A < D B < C B < D
Eating Behavior	26.5 (17.1)	39.1 (18.8)	51.4 (17.2)	49.2 (17.9)	***	0.13	A < B A < C A < D B < C B < D
Mealtime Surroundings	12.3 (13.1)	26.1 (19.7)	47.6 (20.0)	43.5 (20.8)	***	0.24	A < B A < C A < D B < C B < D
Social Situation at Mealtime	34.1 (12.5)	38.3 (11.6)	46.7 (12.2)	45.2 (13.5)	***	0.10	A < C A < D B < C B < D
Disordered Eating	7.55 (10.1)	10.9 (8.72)	13.7 (9.28)	14.5 (9.31)	***	0.04	A < B A < C A < D B < C B < D
Recognizing Hunger/Satiety Cues	27.7 (17.7)	49.3 (16.5)	54.9 (17.8)	55.0 (17.4)	***	0.11	A < B A < C A < D B < C B < D

***p < .001.

Adults with autism-only or autism/ADHD scored significantly higher than comparison participants on all subscales of eating problems. Furthermore, adults with ADHD-only scored higher than comparisons on all subscales except for Purchase of Food and Social Situation at Mealtime. When comparing between diagnostic groups, the autism-only vs autism/ADHD contrast was not significant for any subscale. Moreover, adults with autism-only or autism/ADHD scored higher than adults with ADHD-only on all but one subscale; Motor Control for the autism/ADHD vs ADHD-only contrast was nonsignificant.

### Predictors of eating difficulties

Hierarchical multiple regression revealed that, regardless of diagnosis, total eating difficulties as measured by the SWEAA were positively associated with insistence on sameness, other autistic features, food selectivity, and hyperactivity-impulsivity (see Table 4). Covariates predictive of greater total eating difficulties included younger age, non-binary gender (non-women and non-men), and lifetime eating disorder diagnosis. In a sensitivity analysis with the other autistic features variable rescored to include all 50 items on the AQ, all variables remained significant.

**Table 4.** Hierarchical regression analysis predicting total SWEAA score.

Variable	β	95% CI for β		*SE* β	*p*	*R* ^2^_*Adjusted*_	∆*R*^2^
		LL	UL				
Step 1: Covariates						0.19	38.22***
Age	-0.19	-0.24	0.13	0.03	***		
Women vs. Non-Women	-0.24	-0.31	-0.17	0.04	***		
Men vs. Non-Men	-0.20	-0.27	-0.13	0.04	***		
Medication affecting appetite	-0.06	-0.12	0.00	0.03	*		
Eating Disorder	0.22	0.16	0.28	0.03	***		
Other Mental Illness	0.12	0.06	0.18	0.03	***		
Step 2: Add Variables of Interest						0.65	150.50***
Age	-0.08	-0.12	-0.04	0.02	***		
Women vs. Non-Women	-0.05	-0.10	0.00	0.03	*		
Men vs. Non-Men	-0.06	-0.11	-0.01	0.03	*		
Medication affecting appetite	-0.03	-0.08	0.01	0.02	0.10		
Eating Disorder	0.13	0.08	0.17	0.02	***		
Other Mental Illness	-0.01	-0.05	0.03	0.02	0.69		
Insistence on Sameness	0.45	0.40	0.50	0.03	***		
Other Autistic Features	0.27	0.20	0.34	0.03	***		
Food Selectivity	0.18	0.14	0.22	0.02	***		
Hyperactivity-Impulsivity	0.16	0.10	0.21	0.03	***		
Inattention	0.02	-0.04	0.08	0.03	0.49		

CI = confidence interval; LL = lower limit; UL = upper limit.

*p < .05. ***p < .001.

### Predictors of food selectivity

We also examined food selectivity on the FPQ as a specific type of eating difficulty. Table 5 presents the results of the hierarchical multiple linear regression. Food selectivity was associated with increased scores of eating behavior, perception, other autistic features, and mealtime surroundings, and decreased hyperactivity-impulsivity. A lifetime history of eating disorder diagnosis was positively associated with food selectivity. In a sensitivity analysis with the other autistic features variable rescored to include all 50 items on the AQ, all variables remained significant.

**Table 5.** Hierarchical regression predicting food selectivity score.

Variable	β	95% CI for β		*SE* β	*p*	*R* ^2^_*Adjusted*_	∆*R*^2^
		LL	UL				
Step 1: Covariates						0.05	8.81***
Age	-0.08	-0.14	-0.01	0.03	*		
Women vs. Non-Women	-0.14	-0.21	-0.06	0.04	***		
Men vs. Non-Men	-0.10	-0.17	-0.02	0.04	*		
Medication affecting appetite	-0.07	-0.14	-0.01	0.03	*		
Eating Disorder	0.12	0.06	0.19	0.03	***		
Other Mental Illness	0.05	-0.01	0.12	0.03	0.12		
Step 2: Add Variables of Interest						0.20	19.68***
Age	-0.02	-0.08	0.04	0.03	0.55		
Women vs. Non-Women	-0.04	-0.12	0.03	0.04	0.24		
Men vs. Non-Men	-0.04	-0.11	0.04	0.04	0.33		
Medication affecting appetite	-0.03	-0.09	0.03	0.03	0.35		
Eating Disorder	0.06	0.00	0.12	0.03	*		
Other Mental Illness	0.006	-0.05	0.07	0.03	0.85		
Eating Behavior	0.21	0.13	0.29	0.04	***		
Perception	0.16	0.07	0.25	0.05	***		
Other Autistic Features	0.11	0.04	0.18	0.04	**		
Hyperactivity-Impulsivity	-0.10	-0.17	-0.02	0.04	*		
Mealtime Surroundings	0.10	0.01	0.19	0.05	*		
Purchase of Food	-0.03	-0.10	0.04	0.04	0.40		
Inattention	-0.02	-0.10	0.05	0.04	0.54		

CI = confidence interval; LL = lower limit; UL = upper limit.

* *p* < .05. **p < .01. ***p < .001.

## Discussion

We examined eating difficulties in adults with autism and/or ADHD and further investigated potential mechanisms associated with increased total and specific eating difficulties. To our knowledge, this study is the first to examine the association between eating difficulties and core features of autism and ADHD in adults. There are four main findings. First, individuals with autism-only, ADHD-only, and autism/ADHD had significantly more total (and specific) eating problems than comparisons. Second, autistic participants, with or without ADHD, reported significantly more eating difficulties than all other groups. Third, total eating difficulties were positively associated with insistence on sameness, other autistic features, food selectivity, and hyperactivity-impulsivity. Fourth, food selectivity was positively associated with insistence on sameness, oral sensory sensitivity, and other autistic features, and inversely related to hyperactivity-impulsivity.

Although a number of studies have documented a link between oral sensory sensitivity and food selectivity (e.g. Descrettes-Demey et al., 2023; B. Smith et al., 2020), there has been far less research on the role of cognitive rigidity on eating difficulties. Herein, insistence on sameness predicted increased total eating difficulties and food selectivity. Indeed, insistence on sameness was the strongest partial predictor of total eating difficulties, with the final model explaining 65% of the total variance. Furthermore, Eating Behavior and Mealtime Surroundings—which are essentially measures of insistence on sameness in the mealtime context—and oral sensory sensitivity were independently associated with food selectivity. Oral sensory sensitivity was one of the most common eating difficulties across all diagnostic groups, surpassed only by Purchase of Food. These findings emphasize the importance of both insistence on sameness and sensory sensitivity on eating behavior, supporting firsthand accounts of autistic adults (Park-Cardoso & da Silva, 2022) and quantitative research on food selectivity (Zickgraf et al., 2022).

Despite a scant literature on the prevalence of eating difficulties in ADHD, there have been few studies on how core ADHD symptoms relate to such challenges, and even fewer on adults. Here, hyperactivity-impulsivity was associated with increased total eating problems. Markedly, adults with ADHD struggled more than comparisons with oral sensory sensitivity, motor control, mealtime routines, disordered eating, and recognition of hunger/satiety cues, even when statistically adjusting for medication use. However, in examination of food selectivity, hyperactivity-impulsivity was associated with a *wider* food repertoire, in contrast to prior research (Harris et al., 2022; Zucker et al., 2015). Thus, our results support research linking ADHD to increased total eating problems (Mayes & Zickgraf, 2019) but are inconsistent with the literature on food selectivity and ADHD (Ghanizadeh, 2013; Schwarzlose et al., 2022). A potential explanation is that food selectivity in adults might not be driven by core ADHD features, yet it remains an important issue among individuals with ADHD because subthreshold autistic traits like sensory sensitivity often co-occur (Kochhar et al., 2011; Rani et al., 2023).

A key strength of this study is that we examined both general and specific measures of eating difficulties, supporting a balance of generality and specificity often lacking from the autism eating literature. Specificity is needed to inform the focus of support services and interventions. Still, our study has several limitations. The questionnaires used in this study were written at a sixth-grade reading level, meaning the results may not be generalizable to people with more limited verbal skills. In addition, our recruitment method raises potential for selection bias, as participants are more likely to have a personal relation to the topic. This study’s oversampling of females and non-binary individuals with autism or ADHD may reflect this, given that gender was a significant covariate in this study, and previous research has shown that eating difficulties are more pronounced (and may present differently) among autistic females and gender diverse youth compared to autistic males (Remnélius et al., 2022; Schröder et al., 2022; Simpson et al., 2024; van ‘t Hof et al., 2020). Moreover, we acknowledge that the eating disorder covariate in this study reflected any lifetime history of an eating disorder, rather than a current condition. It is a limitation that data on current and specific types of eating disorders is unavailable. Finally, we were unable to verify formal diagnoses of autism and ADHD, so group differences should be interpreted cautiously. Still, 96% of comparison participants scored below symptom screener cut-offs for autism and ADHD on the AQ and SNAP-IV, respectively. The remaining 4% (*n* = 3) of comparison participants reported other mental illnesses known to impact attention and social functioning, or they had just narrowly exceeded the threshold.

In terms of implications: First, food selectivity in adults may have more to do with a need for sameness than previously recognized. This finding highlights the importance of targeting both rigidity and sensory sensitivity in food selectivity interventions—and suggests that individuals with high food selectivity may have more tolerance for enjoying different foods than often assumed. In addition, individuals with autism or ADHD may be at elevated risk of Avoidant/Restrictive Food Intake Disorder (ARFID), a feeding/eating disorder of extreme food selectivity related to sensory distress or fear of new foods (APA, 2022). In the *DSM-5*,“feeding disorder of infancy or early childhood,” formerly diagnosed in children ages 6 and younger, was replaced by ARFID to capture adolescents and adults with severe restrictive eating not motivated by weight or body image concerns (APA, 2022; Zickgraf et al., 2022). Longitudinal studies are needed to illuminate trajectories throughout the lifespan and understand why some outgrow their eating difficulties while others do not. Moreover, clinicians should screen adults with autism or ADHD for eating problems and eating disorders (Nickel et al., 2019), as results indicate this is an area deserving of attention and support in this population.

In summary, our study confirms that eating is a key challenge for many autistic adults and (to a lesser degree) adults with ADHD. In addition, core features of autism and ADHD were associated with increased total eating difficulties. Notably, insistence on sameness and oral sensory sensitivity predicted increased food selectivity. Additional work is needed to understand the mechanisms of eating difficulties in adults with autism and/or ADHD and improve treatments to address the specific needs of these populations.

## Notes

1. We use both identity-first (e.g. autistic person) and person-first (e.g. person with autism) language to accommodate different preferences within the autism community (Dwyer et al., 2022). This decision is informed by findings that age of diagnosis, experience with stigma, the strength of autism identity, native language, and culture shape the language preferences of autistic adults and parents of people with autism (Buijsman et al., 2023; Bury et al., 2023). Alternatively, person-first language is preferred by many individuals with ADHD (Mackenzie, 2017)—so, we use such language in reference to people with ADHD while acknowledging variability in preferences in this community.
